# Magnetic Resonance Imaging of Cerebral Malaria Patients Reveals Distinct Pathogenetic Processes in Different Parts of the Brain

**DOI:** 10.1128/mSphere.00193-17

**Published:** 2017-06-07

**Authors:** Sanjib Mohanty, Laura A. Benjamin, Megharay Majhi, Premanand Panda, Sam Kampondeni, Praveen K. Sahu, Akshaya Mohanty, Kishore C. Mahanta, Rajyabardhan Pattnaik, Rashmi R. Mohanty, Sonia Joshi, Anita Mohanty, Ian W. Turnbull, Arjen M. Dondorp, Terrie E. Taylor, Samuel C. Wassmer

**Affiliations:** a Center for the Study of Complex Malaria in India, Ispat General Hospital, Rourkela, Odisha, India; b Brain Infections Group, Institute of Infection and Global Health, University of Liverpool, Liverpool, United Kingdom; c Department of Radiology, Ispat General Hospital, Rourkela, Odisha, India; d Department of Radiology, Queen Elizabeth Central Hospital, Blantyre, Malawi; e Infectious Diseases Biology Unit, Institute of Life Sciences, Bhubaneswar, Odisha, India; f Department of Intensive Care, Ispat General Hospital, Rourkela, Odisha, India; g Department of Ophthalmology, Ispat General Hospital, Rourkela, Odisha, India; h North Manchester General Hospital, Manchester, United Kingdom; i Faculty of Tropical Medicine, Mahidol University, Bangkok, Thailand; j Centre for Tropical Medicine and Global Health, Nuffield Department of Clinical Medicine, Oxford, United Kingdom; k Department of Osteopathic Medical Specialties, College of Osteopathic Medicine, Michigan State University, East Lansing, Michigan, USA; l Blantyre Malaria Project, University of Malawi College of Medicine, Blantyre, Malawi; m Department of Immunology and Infection, London School of Hygiene & Tropical Medicine, London, United Kingdom; University at Buffalo

**Keywords:** MRI, PRES, *Plasmodium falciparum*, vasogenic edema, cerebral malaria

## Abstract

The mechanisms underlying the rapidly reversible brain swelling described in patients with cerebral malaria (CM) are unknown. Using a 1.5-Tesla (T) magnetic resonance imaging (MRI) scanner, we undertook an observational study in Rourkela, India, of 11 Indian patients hospitalized with CM and increased brain volume. Among the 11 cases, there were 5 adults and 6 children. All patients had reduced consciousness and various degrees of cortical swelling at baseline. The latter was predominately posterior in distribution. The findings on diffusion-weighted imaging (DWI) and apparent diffusion coefficient (ADC) maps were consistent with vasogenic edema in all cases. Reversibility after 48 to 72 h was observed in >90% of cases. DWI/ADC mismatch suggested the additional presence of cytotoxic edema in the basal nuclei of 5 patients; all of these had perfusion parameters consistent with vascular engorgement and not with ischemic infarcts. Our results suggest that an impairment of the blood-brain barrier is responsible for the brain swelling in CM. In 5 cases, vasogenic edema occurred in conjunction with changes in the basal nuclei consistent with venous congestion, likely to be caused by the sequestration of *Plasmodium falciparum*-infected erythrocytes. While both mechanisms have been individually postulated to play an important role in the development of CM, this is the first demonstration of their concurrent involvement in different parts of the brain. The clinical and radiological characteristics observed in the majority of our patients are consistent with posterior reversible encephalopathy syndrome (PRES), and we show for the first time a high frequency of PRES in the context of CM.

**IMPORTANCE** The pathophysiology and molecular mechanisms underlying cerebral malaria (CM) are still poorly understood. Recent neuroimaging studies demonstrated that brain swelling is a common feature in CM and a major contributor to death in pediatric patients. Consequently, determining the precise mechanisms responsible for this swelling could open new adjunct therapeutic avenues in CM patients. Using an MRI scanner with a higher resolution than the ones used in previous reports, we identified two distinct origins of brain swelling in both adult and pediatric patients from India, occurring in distinct parts of the brain. Our results support the hypothesis that both endothelial dysfunction and microvascular obstruction by *Plasmodium falciparum*-infected erythrocytes make independent contributions to the pathogenesis of CM, providing opportunities for novel therapeutic interventions.

## Abstract (precis)

The pathophysiology and molecular mechanisms underlying cerebral malaria (CM) are still poorly understood. Recent neuroimaging studies demonstrated that brain swelling is a common feature in CM and a major contributor to death in pediatric patients. Consequently, determining the precise mechanisms responsible for this swelling could open new adjunct therapeutic avenues in CM patients. Using an MRI scanner with a higher resolution than the ones used in previous reports, we identified two distinct origins of brain swelling in both adult and pediatric patients from India, occurring in distinct parts of the brain. Our results support the hypothesis that both endothelial dysfunction and microvascular obstruction by *Plasmodium falciparum*-infected erythrocytes make independent contributions to the pathogenesis of CM, providing opportunities for novel therapeutic interventions.

## INTRODUCTION

*Plasmodium falciparum* malaria is a complex disease with a broad spectrum of manifestations. The pathophysiology of cerebral malaria (CM), its most severe form, is still poorly understood. During the past decade, magnetic resonance imaging (MRI) facilities have become increasingly accessible in countries where malaria is endemic, creating new opportunities to investigate the mechanisms underlying the occurrence of CM in living patients (1).

By allowing the comparison of specific parameters between CM patients who survive and those who succumb to the disease, the systematic use of MRI in an extensive study performed in patients from Malawi demonstrated the importance of increased brain volume as a major contributor to death in pediatric CM. The cerebral swelling is transient and quickly reversible with routine treatment in survivors but is associated with progression to herniation and respiratory arrest in fatal cases (2). This general mechanism is consistent with the rapid and complete recovery observed in the majority of surviving cases, and while various hypotheses have been suggested, the cellular pathogenesis underlying the rapidly reversible coma in CM remains unknown (3–5).

In this study, we performed serial brain scans in 11 patients with CM from India who presented with increased brain volume on MRI to investigate the pathophysiology of brain swelling in CM.

## RESULTS

### Clinical course.

**FIG 1:**
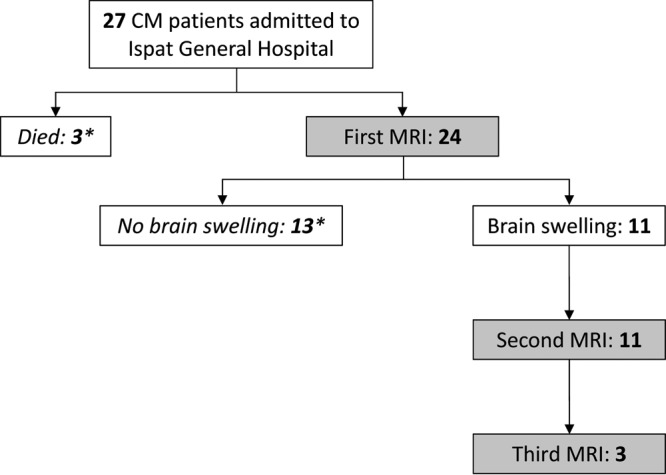
Flowchart of the study. Of the initial 27 patients enrolled in the study, 3 patients died before undergoing MRI and 24 were scanned. Thirteen patients had no brain swelling and were not included in the present analysis. Eleven patients had brain swelling and underwent one (*n* = 11) or two (*n* = 3) additional scans. Patients not included in the study are indicated by an asterisk.

During the study period, 27 patients with CM were admitted to Ispat General Hospital (IGH). Three of the patients died shortly after admission and treatment and did not undergo MRI; 24 survived (88.8%). Eleven of these patients had an MRI abnormality at baseline consistent with increased brain volume and were included in our study. These 11 nonfatal CM cases formed the basis of our analysis (Fig. 1). There were five adult and six pediatric patients. The median (range) age was 29 (22 to 40) years for adults and 12 (5 to 15) years for children. There were 4 (80%) adult and 5 (83.3%) pediatric male cases. All patients were comatose (i.e., Glasgow coma score [GCS] of ≤9 of 15 or Blantyre coma score [BCS] of ≤2 of 5) at baseline. Three of five (60%) adults had a GCS of 15 by 72 h, and all were fully recovered by 1 month, whereas in the pediatric cases, 5/6 (83.3%) had a GCS of 15 by 72 h, and all were fully recovered by 1 month. Overall, rapid recovery was seen in all 11 patients. Key clinical features, complications, and retinal findings are detailed in Table 1. None of the patients had sickle cells. All patients had complete neurological recovery; there were no fatalities.

**TABLE 1:** Summary of the clinical, retinal, and MRI findings in 11 Indian patients with nonfatal CM and with PRES or PRES-like features

Patient group, ID^a^	Age	Sex^b^	Key clinical features (no. of seizures; drug history; BP [mm Hg]; creatinine [mg/dl])^c^	Highest temp (°F)	MRI 1 (10 h)		Clinical features (10 h)^e^				Features of MRI 2 (48–72 h)	Associated clinical features (48–72 h)				Features of MRI 3 (1 mo)	Associated clinical features (1 mo)				Classification
					Features	Type of edema^d^	GCS	Ret	Comp	Parasitemia (parasites/µl)		GCS	Ret	Comp	Parasitemia (parasites/µl)		GCS	Ret	Comp	Parasitemia (parasites/µl)	
Adults																					
6	40	F	2; NA; 120/80; 1.0	99	Marked generalized thickness of cortex in right parieto-occipital region; features of vascular engorgement	Vasogenic edema with some features of ischemia consistent with vascular engorgement	6/15	−	No	291	Nearly complete resolutions of cortical changes	15/15	NP	No	Clear	NP	15/15	NP	No	Clear	PRES-like
17	32	M	2; 0; BP, 120/80; 1.6	98.6	Marked generalized thickening of entire cortical mantle; bilateral effacement of sulci in posterior, temporal, parietal, and occipital lobes	Vasogenic edema^f^	5/15	+ (H [1–5])	Yes (J)	58,178	Nearly complete resolutions of cortical changes	14/15	+ (H [6–20])	Yes (J)	Clear	NP	15/15	−	No	Clear	PRES
22	23	M	0; AM + AP; 110/70; 16.5	99.2	Marked generalized thickness of cortical mantle, right hemisphere involvement greater than left; localized effacement of sulci	Vasogenic edema^f^	9/15	−	Yes (J + AKI)	12,372	Nearly complete resolutions of cortical changes	15/15	NP	Yes (AKI)	Clear	Normal findings	15/15	NP	No	Clear	PRES
28	22	M	2; NA; 110/70; 1.1	100.9	Mild generalized thickening of entire cortical mantle; bilateral focal effacement of sulci in parietal and occipital lobes	Vasogenic edema with some features of ischemia consistent with vascular engorgement affecting BG	5/15	+ (H [1–5])	Yes (J)	12,654	Complete resolutions of cortical changes	14/15	+ (H [1–5])	No	Clear	Normal findings	15/15	−	No	Clear	PRES-like
60	29	M	0; AM; 110/90; 3.2	100	Moderate generalized thickening of entire cortical mantle; right hemisphere involvement greater than left and posterior greater than anterior on each side; generalized sulcal effacement	Vasogenic edema with some features of ischemia consistent with vascular engorgement affecting BG, thalami, and subcortical WM	6/15	−	Yes (J + AKI)	378	Complete resolutions of cortical changes	15/15	NP	Yes (J)	Clear	NP	15/15	NP	No	Clear	PRES-like
Children																					
31	12	M	0; AP; 110/80; 0.5	98.6	Moderate generalized thickening of entire cortical mantle with sulcal effacement of temporal, parietal, and occipital lobes	Vasogenic edema	7/15	−	No	21,931	Partial resolutions of cortical changes	15/15	+ (H [1–5])	No	Clear	NPC	15/15	−	No	Clear	PRES
32	15	M	0; AP; 90/60; 0.5	100	Mild generalized thickening of cortical mantle, posterior greater than anterior	Vasogenic edema	9/15	−	No	203	Partial resolutions of cortical changes	15/15	+ (H [1–5])	No	Clear	NPC	15/15	−	No	Clear	PRES
33	5	M	0; AP; 90/70; 1.3	98.8	Moderate generalized thickening of entire cortical mantle with generalized sulcal effacement, more pronounced in posterior temporal parietal, and occipital lobes	Vasogenic edema^f^	7/15	−	Yes (J)	12,887	No significant improvement	15/15	−	Yes (J + AKI)^f^	Clear	NPC	15/15	NP	No	Clear	PRES
41	11	M	0; AM; 130/110; 0.7	99	Marked generalized cortical thickening of entire cortical mantle, global widespread sulcal effacement	Vasogenic edema with some features of ischemia consistent with vascular engorgement affecting subcortical WM	5/15	−	Yes (J)	14,750	Nearly complete resolutions of cortical changes	15/15	−	Data NA	Clear	Normal findings	15/15	NP	No	Clear	PRES-like
46	12	F	0; AM; 87/60; 0.5	100	Marked generalized cortical thickening of entire cortical mantle, widespread sulcal effacement in posterior temporal parietal, and occipital lobes	Vasogenic edema with some features of ischemia consistent with vascular engorgement affecting subcortical WM and BG (LN)	6/15	+ (H [1–5])	Yes (SA)	163	Nearly complete resolutions of cortical changes	15/15	−	Data NA	Clear	NP	15/15	NP	No	Clear	PRES-like
59	5	M	2; NA; 90/60; 0.7	100.4	Moderate cortical thickening of right frontal, temporal, and parietal lobes, mild cortical thickening of left posterior parietal, and occipital lobes	Vasogenic edema	6/15	−	No	219,097	Nearly complete resolutions of cortical changes	14/15	−	No	200	NP	15/15	NP	No	Clear	PRES-like

a ID, identification number.

b F, female; M, male.

c Drug history, treatment prior to admission; NA, not available; AM, antimalarial drug; AP, antipyretic drug; BP, blood pressure on admission (systolic/diastolic); creatinine, laboratory range is 0.8 to 1.4 mg/dl.

d BG, basal ganglia; WM, white matter; LN, lentiform nucleus.

e GCS, Glasgow coma score; Ret, retinopathies; +, present; −, absent; H, hemorrhages (number of retinal hemorrhages); Comp, complication; J, jaundice; AKI, acute kidney injury; SA, severe anemia; NP, not performed (not necessary); NPC, not performed (conscious and noncooperative pediatric patients).

f Perfusion, CBV, CBF, and MTT were not available.

### MRI findings.

**FIG 2:**
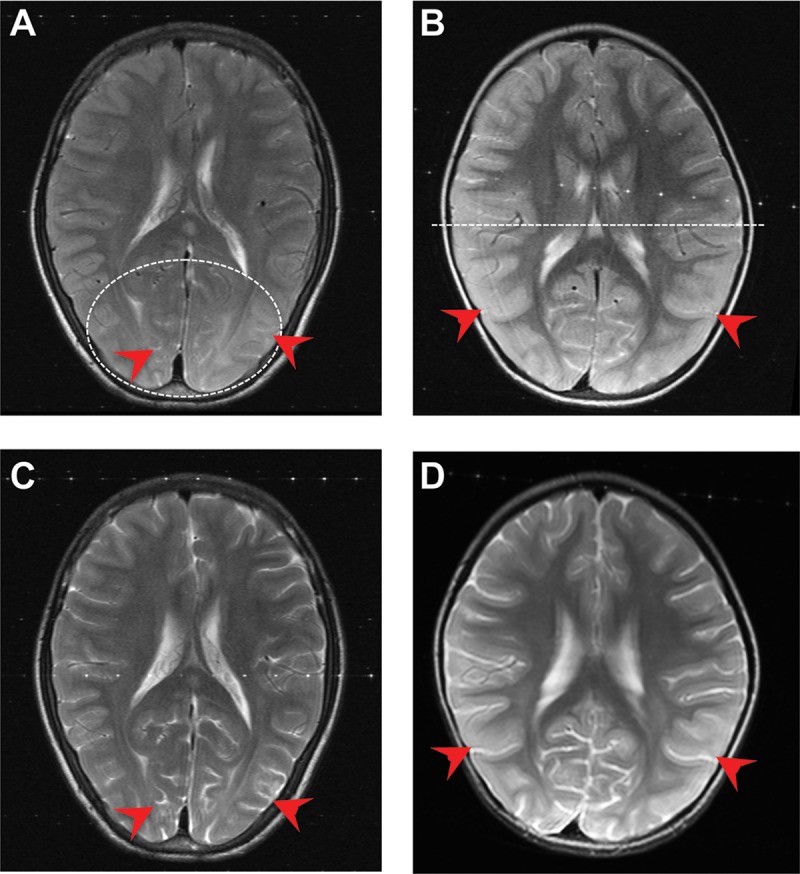
Cases ID32 (left) and ID46 (right). (A, B) Axial T2 images demonstrate a marked cortical thickening of the entire cortical mantle in both patients, with widespread sulcal effacement in the posterior temporal parietal and occipital lobes (red arrows). (C, D) Interval axial T2 images showing nearly complete resolution of the cortical swelling 48 to 72 h postadmission and treatment, with visible sulci (red arrows).

All 11 cases in our series had generalized swelling (involving >2 lobes); the swelling was marked in 5/11 (45%) patients, moderate in 4/11 (36%), and mild in 2/11 (18%). All 11 had cortical thickening, which was either unilateral (2/11, 18%) or bilateral (9/11, 81.8%). An increased signal on axial T2-weighted and fluid-attenuated inversion recovery (T2/FLAIR) was present in all cases during the first scan, and in 8/11 (72.7%) cases, the distribution was posterior, involving the occipital/parietal and temporal lobes and sparing the frontal lobe (Fig. 2A and B). After 48 to 72 h, complete or nearly complete resolution of cortical thickening and T2/FLAIR signal was seen in all the adult cases and 3/6 (50%) pediatric cases (Fig. 2C and D). Among the latter group, the three remaining patients did not undergo an additional scan but remained clinically well (Table 1).

### (i) Vasogenic and cytotoxic edema.

**FIG 3:**
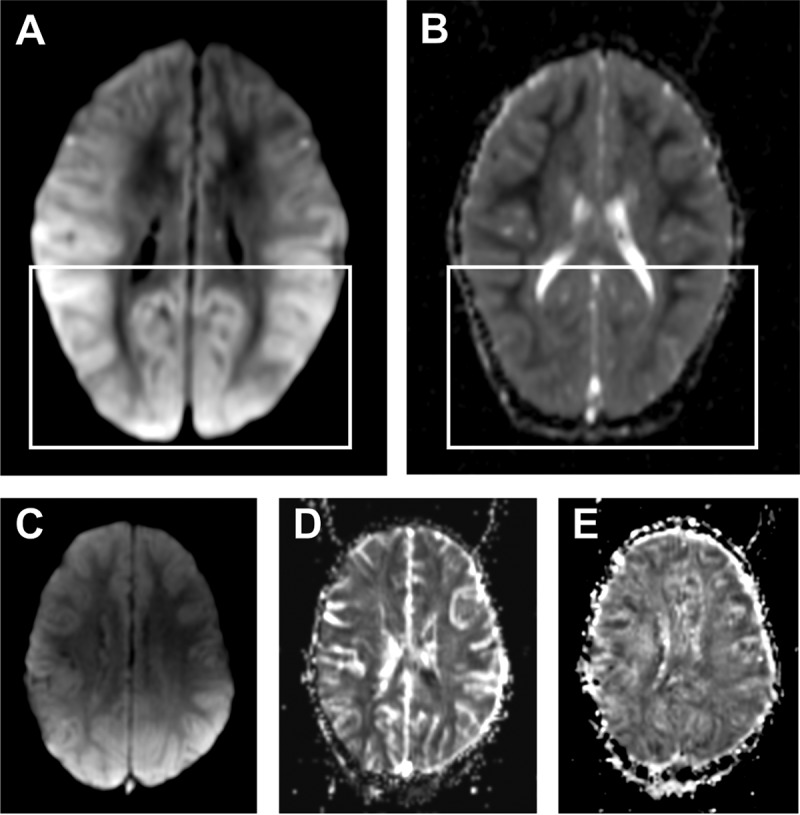
Case ID41. Increased DWI signal in the cortex on admission, with posterior predominance (A) and no mismatch in corresponding ADC image (B), consistent with vasogenic edema (white boxes). Cortical increase of CBF (C) and CBV (D) with minimal reduction of MTT (E) in the same area.

**FIG 4:**
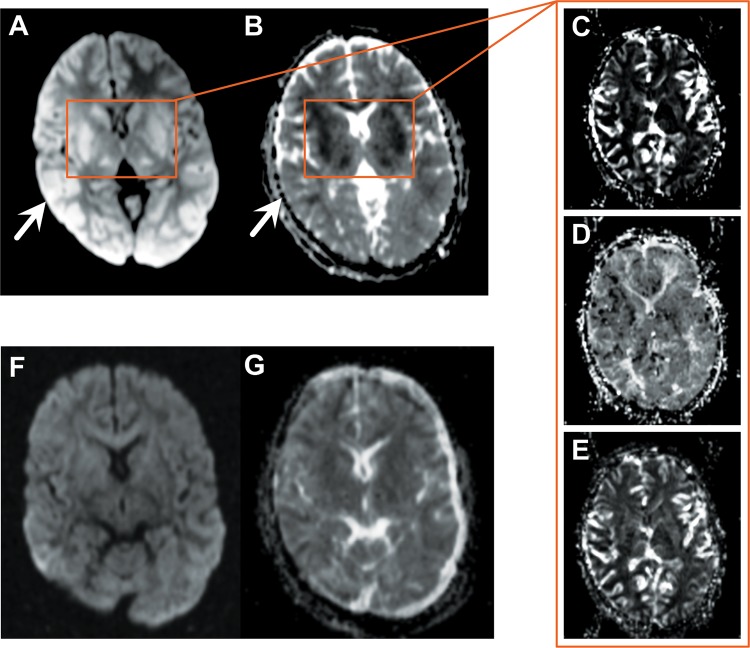
Case ID60. (A to E) Increased DWI signal in the posterior cortex (A, white arrow) with no mismatch in corresponding ADC image (B, white arrow). DWI and ADC mismatch in the basal ganglia (A and B, orange boxes) due to vascular engorgement demonstrated by an increased CBV (C), slightly delayed MTT (D), and nearly normal CBF (E). (F, G) Interval DWI image (F) and corresponding ADC map (G) demonstrating a complete resolution of the lesions 48 to 72 h postadmission and treatment.

There was evidence of vasogenic edema in all 11 patients scanned (Fig. 3 and 4A and B). The severity of vasogenic edema was variable and accounted for the degree of brain swelling described above. In five patients, there were patchy areas of diffusion-weighted imaging/apparent diffusion coefficient (DWI/ADC) mismatch, consistent with ischemia and cytotoxic edema. Cerebral blood volume (CBV), cerebral blood flow (CBF), and mean transit time (MTT) were also captured, and the kinetics of perfusion were analyzed on a regional basis. In these patients, the increased CBV, minimally delayed CBF, and slow MTT were consistent with vascular engorgement (Fig. 4C to E). In all cases, both features reversed rapidly upon treatment (Fig. 4F and G).

### (ii) PRES.

Because of the posterior predominance of the radiological features and the rapid clinical improvement following antimalarial treatment, we sought to explore in more detail the features of posterior reversible encephalopathy syndrome (PRES) in these cases. All had vasogenic edema, and the imaging abnormalities were mostly posterior, as described above. The changes were predominately cortical in 7/11 (63.7%) patients and infrequently involved the subcortical regions (4/11, 36.3%). Nine of the 11 (82%) had bilateral changes, while two were strictly unilateral. Many of the features discussed above are consistent with PRES. However, the presence of vascular engorgement observed in five patients (cases ID6, ID28, ID60, ID41, and ID46) is not consistent, and thus, the term PRES-like was used. No patients had high blood pressure or were taking drugs associated with PRES prior to their admission (Table 1). Two adult patients had renal impairment.

### Malarial retinopathy.

Retinal hemorrhages were observed in 5 of the 11 patients (45%). Three patients had hemorrhages on admission, which were reversible within 48 to 72 h in one patient, stayed unchanged in another patient, and worsened in the third. Two patients developed retinal hemorrhages 48 to 72 h postadmission. Hemorrhages were resolved in all patients at follow-up (Table 1). No other features of retinopathy were observed.

## DISCUSSION

In a comprehensive MRI analysis of 11 nonfatal pediatric and adult CM cases with increased brain volume, we report observations consistent with vasogenic edema, involving the posterior part of the brain in all cases. Five of these patients had concomitant MRI signatures of vascular congestion in their basal nuclei, reflecting involvement of the deep venous system and its tributaries. All patients presented clinical and radiological evidence of rapid reversibility after antimalarial treatment. Some of the changes we report here are hallmarks of posterior reversible encephalopathy syndrome (PRES) and suggest that pathogenetic mechanisms in CM may induce PRES or a PRES-like signature on MRI.

The etiology of brain swelling in CM is unknown but likely to be multifactorial. The microvascular pathology of this neurologic syndrome is unique and mainly caused by the binding of *P. falciparum*-infected erythrocytes (IEs) to vascular endothelium, leading to microvascular obstruction. This phenomenon is called sequestration and is significantly and quantitatively linked to premortem coma in CM patients (6). Adhesive forces causing clumping of parasitized (autoagglutination) and uninfected red cells (rosetting), as well as reduced red cell deformability, are thought to obstruct further microcirculatory flow. In addition, by their adherence to microvascular beds, IEs also induce a broad range of both pathogenetic and protective responses, as well as endothelial activation and signaling. The latter can lead to blood-brain barrier (BBB) leakage, a process previously described in CM patients with various degrees of severity (7–11). Here, we report for the first time that in CM patients with brain swelling, BBB dysfunction occurs in mainly posterior areas of the brain, both in adults and children. The presence and reversibility of retinal hemorrhages in some of our patients did not mirror their cerebral features on MRI, and additional studies are needed to assess the potential correlation between cerebral vascular impairment and retinal hemorrhages in a larger Indian cohort. The local loss of BBB function leads to vasogenic edema, resulting from the plasma leaking into the brain parenchyma. However, evidence for a generalized increase in BBB permeability in CM is still debated (9), and the global distribution of vasogenic edema was only observed in one of our patients. Plugging of microvessels by IE sequestration (12), platelet accretion (13), fibrin thrombi (14), red blood cells with reduced deformability (15), rosetting (16), or a combination of the above (17) can contribute to parenchymal hypoperfusion, as well as to BBB dysfunction from increasing local hypertension. Augmented filtration pressure at the level of the capillary beds could overcome the vascular endothelium and tight intercellular junctions of the BBB, resulting in endothelial dysfunction and surrounding vasogenic edema.

There was evidence of cytotoxic edema (i.e., DWI and ADC mismatch) in 5 of 11 patients, and in this group, the perfusion parameters were more consistent with vascular engorgement than an ischemic event *per se*. Vascular engorgement is an increase in blood volume, which can be caused by arterial vasodilatation or obstruction of the cerebral veins and venous sinuses (18). The intravascular biomass of sequestered IEs may explain the microvascular dilatation and engorgement observed in these patients, thereby contributing to the increase in cerebral volume seen on imaging. This is in line with the positive correlation between microvascular engorgement and coma reported in CM (6). The occurrence of vascular engorgement on MRI was mainly seen in the basal nuclei in our CM cohort. Previous studies showed that these deep and highly vascularized structures of the brain are prone to lacunar strokes, as their penetrating arteries are small and branch directly off a larger, high-pressure, heavily muscled main artery without tapering (19). Such small penetrating arteries may also be particularly vulnerable to IE sequestration, and the resulting decreased perfusion could explain the vascular engorgement of the basal nuclei.

Some of the clinical and radiological characteristics observed in our cohort, namely, the presence of vasogenic edema with posterior presentation and fast reversibility upon treatment, suggest a PRES-like phenomenon. PRES is a clinicoradiologic disorder of reversible cortical and/or subcortical brain edema in patients with acute neurological symptoms (seizures, encephalopathy, visual disturbances, and alterations of consciousness) in the setting of a broad range of disorders, including bacterial infection (20). MRI correlates typically include vasogenic edema in the cortex, subcortex, and white matter of the parieto-occipital lobes bilaterally (21); frontal lobe and cerebellar involvement are less frequent (22). A key element of the diagnosis of PRES is the reversibility, which is usually associated with a favorable outcome. However, in its severe form, PRES can be associated with death and disability; posterior fossa edema causing obstructive hydrocephalus is one of the postulated causes of persisting sequelae and death (20, 23).

Although the pathogenesis of PRES remains unclear, impaired cerebral autoregulation caused by the presence of hypertension, drugs (e.g., cyclosporine), or bacterial infections is thought to increase cerebral perfusion, endothelial dysfunction, and breakdown of the BBB, resulting in vasogenic edema (20). These processes are consistent with the disease mechanisms described in CM, and the high frequency of PRES-like presentations we report suggests that an endotheliopathy may be contributing to the pathophysiology of CM in a subset of patients. The predilection for involvement of posterior circulation territories in PRES is suspected to result from the relatively sparse sympathetic innervation of the posterior circulation (24). As sympathetic stimulation is vasoprotective in both hypertensive (25) and endothelial inflammatory (26) causes of PRES, this sympathetic neural network asymmetry would explain why the BBB is more susceptible to dysfunction caused by IE sequestration and subsequent inflammation in the posterior areas during CM. This anterior/posterior difference may have influenced the variability in BBB disruption reported in previous CM histopathology studies, depending on the areas of postmortem brain sampling.

Our understanding of PRES, both in terms of pathogenesis and phenotype, is still evolving (20). Malaria has only been reported as a cause of PRES in one isolated case (27). In the absence of other causes, the underlying etiology of PRES in our series is the *P. falciparum* infection. While acute kidney injury is commonly present in patients with PRES or severe malaria, this complication was only observed in two patients and cannot account for the changes observed in the rest of the cohort. Our systematic study of patients in this setting where malaria is endemic underscores for the first time a high frequency of a PRES-like presentation in CM, affecting both adults and children. While cortical involvement with posterior predominance on DWI images was described in some patients from Malawi (2, 28), the lack of ADC maps did not allow the identification of vasogenic edema and, therefore, of PRES. The presence of radiologic signatures of vasogenic edema alone or in conjunction with vascular engorgement in the majority of our patients implicates endothelial dysfunction and sequestration of IEs in the pathogenesis of CM, a theory that was suggested previously (29).

The present study has several limitations. First, it is a small, single-center series of case studies, and further analyses involving higher patient numbers are warranted. Second, we selected 11 of 24 patients with CM based on the presence of abnormalities on MRI, and the mechanisms of coma are likely to be more diverse than described here. In addition, the patient selection was restricted to clinically stable subjects who were able to undergo lengthy MRI scans. Consequently, our results are limited to nonfatal CM, where PRES may correlate with a better outcome. It is plausible that different pathogenetic processes are involved in fatal cases, which could explain the lack of benefit from mannitol, an osmotic diuretic that lowers intracranial pressure by absorbing extracellular fluid into brain capillaries, as an adjunctive therapy in CM (10, 30). Indeed, it is conceivable that ischemic brain injury occurs in fatal cases (31) and develops secondarily after vasogenic edema. Further studies are under way to investigate this hypothesis. Third, although our analysis was focused on patients with changes on baseline MRI, it is possible that the excluded cases may have had subtle changes. Future investigations should consider this cohort to facilitate risk stratification on prognosis. Finally, without angiographic imaging, we were unable to describe the involvement of the vasculature.

In conclusion, our results give new insight into the pathophysiology of reversible brain swelling frequently observed in CM and corroborate the hypotheses suggesting that an impaired BBB and vascular engorgement are contributors to the increased brain volume seen in CM patients. A better understanding of the disease mechanisms and risk stratification on prognosis may provide guidance toward new interventions.

## MATERIALS AND METHODS

### Study site and patients.

The study was carried out at Ispat General Hospital (IGH) in Rourkela, in the state of Odisha, India, from October 2013 to August 2015. All patients satisfied a strict definition of CM according to the modified World Health Organization criteria. Consecutive CM patients with coma (defined as a Glasgow coma score [GCS] of ≤9 out of 15 for adults and a Blantyre coma score [BCS] of ≤2 for young children) after correction of hypoglycemia (<2.2 mmol/liter) and infected with *Plasmodium falciparum* (detected by rapid diagnostic test and confirmed by the presence of asexual forms of the parasite in a peripheral blood smear) were eligible for inclusion. Patients who were clinically unstable because of shock (systolic blood pressure of <80 mm Hg with cool extremities) or signs of respiratory insufficiency (respiratory rate above 40/min, nailbed oxygen saturation <90% by pulse oximetry) were excluded because of the increased risk incurred by being transported to the MRI facilities. Other exclusion criteria included the presence of coinfection by other plasmodial species detected by either rapid diagnostic test or peripheral blood smear examination, metallic devices, documented allergies to MRI contrast media, a diagnosis of meningitis or other causes of encephalopathy, and in female patients, pregnancy or lactation. Patients whose relatives did not consent to enrollment or who died within 48 h of admission or regained consciousness before imaging were excluded. Patients with an underlying bacterial infection were also excluded, on the basis of clinical observations and positive blood, urine, or cerebrospinal fluid culture.

Due to the lack of direct association between the presence of retinopathies and CM in low-malaria-transmission settings, retinal changes were documented but not used as a diagnosis parameter in the present cohort. Tonic-clonic seizures were diagnosed based on stereotypical signs and recorded.

### Ethics statement.

Ethical approval was obtained from The Indian Council of Medical Research (TDR589/2010/ECDII), as well as from the institutional review boards of Ispat General Hospital, New York University School of Medicine (S12-03016), and the London School of Hygiene and Tropical Medicine. Because CM patients are comatose, written informed consent was obtained from the families of all patients (five adults and six children) before enrollment in the study. Following our Institutional Review Board (IRB)-approved clinical protocol, a nurse carefully described the study to the potential participants’ families in Oriya, the local language in the state of Odisha. The IRB-approved consent form was then read in its entirety to the families before written consent was sought through signature or inked fingerprint. The nurse in charge of the enrollment and an impartial witness cosigned the form. Patients whose families declined the enrollment were not included in the study. Patient consent has been archived with the authors and is available upon request.

### Study procedures.

On admission, a full medical history and physical examination were conducted and recorded on a standardized clinical record form. Rapid diagnostic tests for the detection of *Plasmodium falciparum* histidine-rich protein II were used for all patients (SD Bioline; Standard Diagnostics, India). Blood samples were collected for complete blood count, parasite count, hemoglobin, hematocrit, glucose, and biochemistry. Other investigations, including ECG and blood culture, were performed when clinically indicated (Table 2). Blood gases were monitored frequently in patients receiving ventilatory support until they recovered.

**TABLE 2:** Summary of the hematological, biochemical, and other findings in 11 Indian patients with nonfatal CM

Patient group, ID^a^			Hematology^b^				Biochemistry^c^										Other finding	
	Wt (kg)	Height (cm)	Hb (g%)	HCT (%)	PLT (×1,000/µl)	WBC (×1,000/µl)	Glucose (mg/dl)	Bilirubin (mg/dl)	GPT/ALT (IU/liter)	Creatinine (mg/dl)	Urea (mg/dl)	Na (mM/liter)	K (mM/liter)	pH	HCO_3_ (mM/liter)	ANA gap (mM/liter)	Blood culture	ECG^d^
Adults																		
6	48	154	10.8	30.3	610	29.1	91	0.2	ND	1	39	142	3.8	ND	ND	ND	ND	ND
17	58	162	10.9	19.3	35	5.3	108	14.2	ND	1.6	149	136	4.7	ND	ND	ND	ND	Normal
22	58	158	14.1	29	28	10.9	100	3.5	107	16.5	117	139	4	7.33	20.2	14	Sterile	ND
28	54	158	10	NA	40	7.4	150	3.3	25	1.1	72	130	4.9	ND	ND	ND	ND	Normal
60	67	155	7.8	24.4	30	15.1	389	6.8	34	3.2	217	131	4.5	ND	ND	ND	ND	ND
Children																		
31	23	142	6.6	NA	62	9.1	138	ND	21	0.5	ND	128	3.2	ND	ND	ND	ND	ND
32	25	142	7.5	NA	102	4.5	ND	ND	24	0.5	ND	135	3.4	ND	ND	ND	ND	ND
33	13	98	10.6	NA	NA	9.8	99	4.5	109	3.3	149	141	3.5	ND	ND	ND	ND	ND
41	22	120	13.6	33.9	44	12.5	210	3.6	90	0.7	76	133	3.6	7.4	27.4	ND	Sterile	Normal
46	12	40	3.2	8.6	25	27.2	ND	2	29	0.5	64	131	5.2	ND	ND	ND	ND	ND
59	12	80	6.3	19.6	26	25.4	10	ND	ND	0.7	47	134	4.8	ND	ND	ND	Sterile	ND

a ID, identification number.

b Hb, hemoglobin; HCT, hematocrit; PLT, platelets; WBC, total white blood cells; NA, not available.

c GPT/ALT, serum glutamic pyruvic transaminase/alanine transaminase; Na, sodium; K, potassium; HCO_3_, serum bicarbonate; ANA gap, anion gap; ND, not done as not requested by the physician in charge.

d ECG, electrocardiogram.

### Retinal examination.

To compare potential differences in pathogenetic processes between retinal and cerebral vasculatures in our cohort of CM cases (32), all patients underwent retinal examination by direct and indirect ophthalmoscopy within 6 h of admission, and the severity of the findings was graded by two ophthalmologists (R. R. Mohanty and S. Joshi) according to published classification criteria (33).

### MR imaging.

Imaging of the brain was performed using a 1.5-Tesla (T) Siemens Symphony MRI scanner (Siemens AG, Erlangen, Germany). Scanning was carried out initially within 10 h of admission and then again between 48 and 72 h after admission. When MRI abnormalities were identified on the second interval scan, a third scan was performed at approximately 1 month. The MRI sequences included sagittal T1-weighted images, axial T2-weighted and fluid-attenuated inversion recovery (T2/FLAIR) turbo spin echo, susceptibility-weighted imaging (SWI), axial trace diffusion-weighted imaging (DWI) (b values, indicative of the degree of diffusion weighting applied, of 0, 500, and 1,000 s/mm^2^), and axial T1 spin echo (T1-SE) after contrast (529 mg/ml gadobenate dimeglumine [Multihance]; Bracco Diagnostics). Contrast injection was performed and monitored by automated injector (MedRad Spectris Solaris EP; Bayer). The pulse sequences included T2, FLAIR, T1, and gradient echo. Apparent diffusion coefficient (ADC) maps were generated and used to confirm restricted diffusion (34). Perfusion parameters, such as cerebral blood volume (CBV), cerebral blood flow (CBF), and mean transit time (MTT), were calculated using previously described techniques (35). From these sequences, we were able to locate and characterize lesions in the brain, describe their physiological features, and identify temporal changes. The degree of generalized brain swelling was semiquantified as mild (just discernible), moderate (clearly evident without mass effect), or marked (extensive with mass effect). Vasogenic edema was defined radiologically as an increased DWI signal with markedly increased diffusion coefficients compared with those of normal white matter (36), whereas cytotoxic edema was defined as DWI hyperintensity associated with decreased diffusion coefficients compared with those of white matter (DWI/ADC mismatch) (37).

### MRI interpretation.

Each MRI was interpreted by two radiologists on-site (M. Majhi and P. Panda) and one experienced radiologist off-site (S. Kampondeni) using NeuroIndia, a searchable database based on a systematic scoring system of brain MRI interpretation derived from NeuroInterp (38). The MR studies and data derived for CBV, CBF, and MTT were analyzed blindly by an independent neuroradiologist off-site (I. W. Turnbull). Discrepant cases were settled by consensus.

### Clinical care.

All patients were treated with intravenous artesunate (2.4 mg/kg of body weight). The first dose was given immediately after diagnosis and the second and third at 12-h intervals, for a minimum of 3 doses. Subsequent doses were administered daily. Once the patients were conscious and able to take medication orally, the treatment was switched to an artemisinin-based combination therapy: oral artesunate (4 mg/kg of body weight) once daily for 3 days together with a single dose of oral sulfadoxine-pyrimethamine (25 and 1.25 mg/kg of body weight) on the first day of oral therapy. A single gametocytocidal dose of primaquine (0.75 mg/kg of body weight) was given on the second day of oral therapy. These treatments were in accordance with the national drug policy of the Government of India. Disability at discharge or new/progression of symptoms by 1 month were determined and described on the basis of the Glasgow or Blantyre coma score, retinal changes, and cranial nerve and motor system function. When possible, a third MRI scan was performed for patients who had magnetic resonance abnormalities on the second scan.
